# A Smart Computational Tool for Personalized Coronary Blood Flow Settings During Normothermic *Ex Situ* Heart Perfusion

**DOI:** 10.1097/MAT.0000000000002394

**Published:** 2025-02-20

**Authors:** Jorik H. Amesz, Niki L. Lupgens, Dirk J. Duncker, Lisa E. Sluijter-Rozendaal, Dwight Dumay, Olivier C. Manintveld, Yannick J. H. J. Taverne

**Affiliations:** From the * Translational Cardiothoracic Surgery Research Lab, Department of Cardiothoracic Surgery, Erasmus University Medical Center, Rotterdam, the Netherlands; † Experimental Cardiology, Department of Cardiology, Erasmus University Medical Center, Rotterdam, the Netherlands; ‡ Departments of Clinical Perfusion and Cardiothoracic Surgery, Erasmus University Medical Center, Rotterdam, the Netherlands; § Department of Cardiology, Erasmus University Medical Center, Rotterdam, the Netherlands; ¶ Erasmus MC Transplant Institute, Erasmus University Medical Center, Rotterdam, the Netherlands.

**Keywords:** normothermic machine perfusion, heart transplantation, coronary physiology

## Abstract

Myocardial edema significantly develops during current subnormothermic *ex situ* heart perfusion (ESHP) procedures, resulting in myocardial function decline during prolonged perfusion. A relatively high coronary blood flow (CBF) during ESHP is thought to be responsible for this high degree of myocardial edema formation. In this study, we present a novel tool to calculate CBF based on individual donor (sex and body weight) and perfusate (hemoglobin concentration, oxygen saturation, partial pressure of oxygen [PO_2_]) characteristics. The tool continuously evaluates the balance between myocardial oxygen consumption (MVO_2_) and delivery to facilitate adequate and preventing excess perfusion. Taking this personalized approach, the CBF can potentially be lowered while still providing sufficient oxygen to the donor heart. Furthermore, the tool automatically calculates MVO_2_, ΔPO_2_, and coronary vascular resistance during ESHP, which aids in the qualitative assessment of the heart before transplantation.

## Graphical abstract

**Figure:**
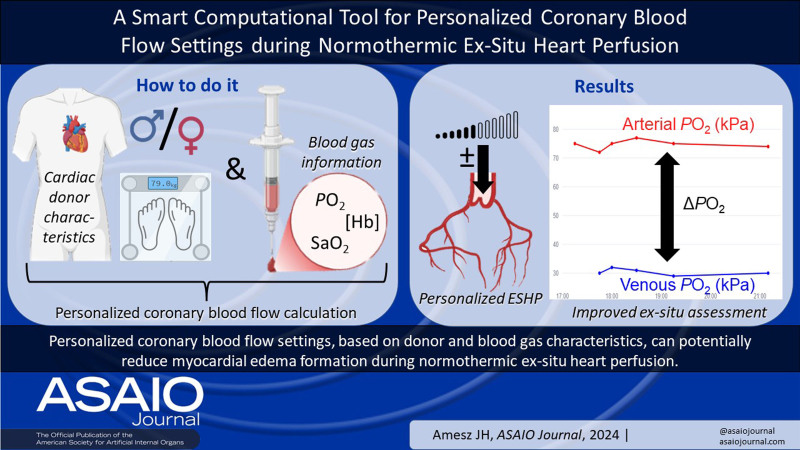

*Ex situ* heart perfusion (ESHP) for cardiac transplantation using the Organ Care System (OCS) (Transmedics, Andover, MA) has been successful in enlarging the cardiac donor pool.^1^ The OCS platform is based on subnormothermic (34°C) Langendorff perfusion, resulting in the preservation of the heart in a beating state.^2^ Aerobic respiration forms the basis of contractile function, thus requiring a tight balance between myocardial oxygen consumption (MVO_2_) of the donor heart and myocardial oxygen delivery (MDO_2_) by the OCS.^3^ The MDO_2_ is determined by coronary blood flow (CBF)^4^ and the perfusate’s oxygen content, where the current OCS protocol prescribes a CBF of 700–800 ml·min^−1^.^2^ This CBF is relatively high as compared to 250–450 ml·min^−1^ in a typical adult resting heart.^4,5^ The current OCS protocol does not allow for CBF settings based on the anatomy of an individual donor, whereas CBF widely varies between individuals *in vivo*. Thus, the heart of a heavy male donor would logically require a higher CBF compared to the heart of a small female donor. Yet, supranormal levels of CBF are associated with myocardial edema formation, resulting in impaired contractility of the donor heart^6^ and potentially primary graft dysfunction (PGD).^7^ Therefore, it is important to prevent excess CBF while still providing sufficient oxygen delivery to the heart. We have previously shown that CBF can be lowered due to excessive MDO_2_ in most OCS runs, yet additional tools are needed to calculate the right MDO_2_ to balance MVO_2_ of the donor heart.^5^ The purpose of this article is to present a novel tool that calculates personalized CBF settings for adequate MDO_2_ on ESHP while minimizing myocardial edema formation. In addition, the tool enables a more detailed metabolic assessment of hearts on ESHP by continuously evaluating the MVO_2_–MDO_2_ balance.

## Materials and Methods

### Standard Organ Care System Settings

The standard OCS settings are a CBF of 700–800 ml·min^−1^, a target aortic pressure of 75–80 mm Hg, and a temperature of 34°C.^2^ The OCS perfusate is a mixture of ≥1,200 ml donor blood, 500 ml of crystalloid priming solution, and 100 ml albumin. Gas flow on the OCS is 150 ml·min^−1^ with a custom gas mixture (85% oxygen, 1% carbon dioxide).^2^

### Rationale for Personalized Coronary Blood Flow Formula

Myocardial oxygen consumption depends on heart rate, contractility, ventricular wall tension, and number of cardiac cells,^3,4^ and can be calculated based on Equation 1. The arterial oxygen content (CaO_2_) is dependent on the hemoglobin concentration ([Hb]), arterial oxygen saturation (SaO_2_), and partial pressure of oxygen (PaO_2_) (Equation 2). The oxygen extraction ratio (OER) of cardiomyocytes is 70–80%.^4^


$$\begin{array}{l} MVO_{2}=CBF\times CaO_{2}\times OER \end{array}$$ (1)

$$\begin{array}{l} CaO_{2}=1.34\times \left[Hb\right]\times SaO_{2}+0.0031\times PaO_{2} \end{array}$$ (2)

Donor hearts on the OCS are assumed to have minor variations in contractility and wall tension and heart rates of approximately 65–80 bpm. Hence, the number of cardiac cells is the primary factor determining the MVO_2_ of hearts on the OCS, which can be derived from the heart weight (HW). Measurement of HW is not standard for heart transplantation procedures but can be estimated from the body weight (BW) and sex of the donor (see Supplementary Equations 1 and 2, Supplemental Digital Content, http://links.lww.com/ASAIO/B422). Myocardial oxygen consumption was expected to be 0.08–0.13 ml·min^−1^ per gram myocardium under subnormothermic resting conditions.^4^ Next, Equation 1 was rewritten to isolate the required CBF, resulting in Equation 3 for personalized CBF.


$$\begin{array}{l} \begin{array}{llll} CBF & =\frac{MVO_{2}}{CaO_{2}\times OER} & =\frac{HW}{1.34\times \left[Hb\right]\times SaO_{2}+0.0031\times PaO_{2}} & \times \frac{MvO_{2}perHW}{OER} \end{array} \end{array}$$ (3)

Application of Equation 3 yields a CBF of approximately 350 ml·min^−1^, which is in accordance with physiologic values^4^ but lower than prevailing clinical OCS settings.^2^ Preclinical studies have demonstrated adequate perfusion with CBF of 500 ml·min^−1^ during ESHP.^6^ Therefore, Equation 3 was normalized between 500 and 800 ml·min^−1^ using Supplementary Equation 3, and extreme values depicted in Supplementary Table 1 (Supplemental Digital Content, http://links.lww.com/ASAIO/B422), coming from experience with ESHP in our center.^5^ This results in Equations 4 and 5 to calculate personalized CBF settings for female (CBF_F_) and male (CBF_M_) donors, respectively (see Supplement C, Supplemental Digital Content, http://links.lww.com/ASAIO/B422).


$$\begin{array}{l} \begin{array}{lll} & CBF_{F}\left(mL\cdot min^{-1}\right)=300 & \;\times \frac{\frac{2.153\times BW+104.6}{1.34\times Hb\times SaO_{2}+0.0031*PaO_{2}}\times 12.5-133}{333}+500 \end{array} \end{array}$$ (4)

$$\begin{array}{l} \begin{array}{lll} & CBF_{M}\left(mL\cdot min^{-1}\right)=300 & \;\times \frac{\frac{2.492\times BW+141.2}{1.34\times Hb\times SaO_{2}+0.0031*PaO_{2}}\times 12.5-178}{431}+500 \end{array} \end{array}$$ (5)

## Results

Based on the presented formulas, a tool with CBF calculator was designed for computation of personalized machine perfusion (PMP) settings. This tool is available for open access at https://personalizedmachineperfusion.streamlit.app/.

**Figure 1.**
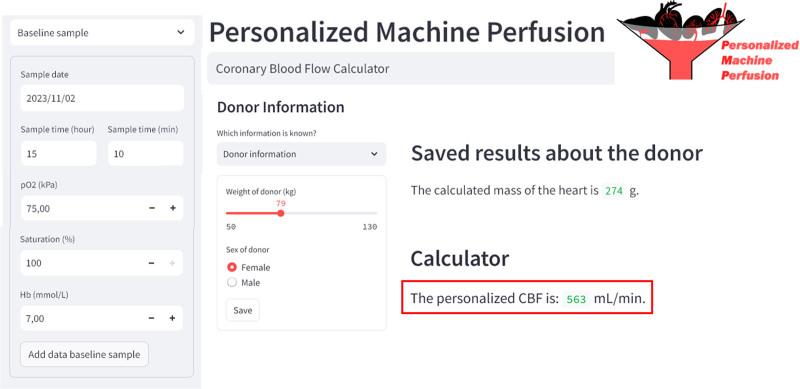
Output of CBF calculator with personalized CBF based on donor characteristics (weight and sex of donor) and blood gas results (PO_2_, oxygen saturation, and [Hb]) from a baseline blood sample. CBF, coronary blood flow; [Hb], concentration of hemoglobin; PO_2_, partial pressure of oxygen.

In the “Donor Information” panel in the tool, donor BW and sex should be entered to calculate the HW or the exact HW can be filled in if measured. Furthermore, the PaO_2_, [Hb], and SaO_2_ values from the first baseline blood sample on OCS should be entered. Subsequently, the tool calculates a personalized CBF as shown in the example of Figure 1, which can be applied for each individual donor heart on the OCS.

**Figure 2.**
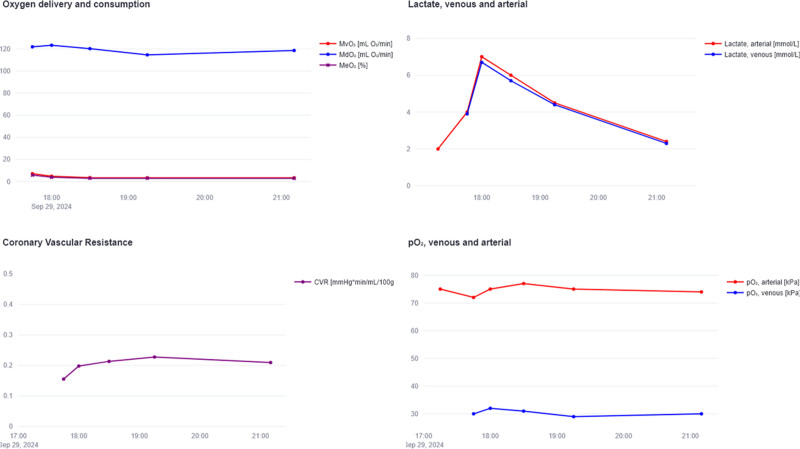
Exemplary graphical presentation of metabolic assessment parameters in the personalized machine perfusion tool during a clinical case. MVO_2_, MDO_2_, MEO_2_, CVR, and PO_2_ are displayed next to lactate trends. CBF, coronary blood flow; CVR, coronary vascular resistance; [Hb], concentration of hemoglobin; MDO_2_, myocardial oxygen delivery; MEO_2_, myocardial oxygen extraction; MVO_2_, myocardial oxygen consumption; PO_2_, partial pressure of oxygen.

Next, subsequent arterial and venous samples can be entered, which updates the CBF calculation according to the actual MVO_2_ of the donor heart. In addition, MVO_2_ trends and other metabolic parameters, *e.g*., lactate, ΔPO_2_, and coronary vascular resistance (CVR), are displayed to facilitate improved assessment of the metabolic function of the heart (Figure 2). Also, electrolyte information can be entered in the “Electrolytes” panel, providing an advice to supplement electrolytes, *e.g*., calcium or bicarbonate, when indicated.

## Discussion

Here, we present a novel tool to compute CBF settings based on individual donor characteristics and MVO_2_ rates during subnormothermic ESHP. As such, donor hearts can be perfused with more personalized CBF settings, potentially minimizing chances of edema formation during ESHP. In addition, real-time feedback on oxygen handling allows for better assessment of graft performance, especially when lactate levels are increasing. Next to that, an advice on electrolyte supplementation is incorporated.

### Personalized Coronary Blood Flow Settings

Cardiac function during ESHP is dependent on adequate CBF. The current OCS protocol depicts a CBF which is 2–3 times higher than normal resting CBF.^4^ Conversely, the MVO_2_ of hearts on the OCS is relatively low due to an unloaded left ventricle and temperature of 34°C.^3^ Together with the high oxygen content of the gas mixture on the device, this typically results in a supranormal MDO_2_ and suggests superfluity of such high CBF. Furthermore, excessive CBF might even contribute to more myocardial edema formation,^6^ associated with myocardial function decline during ESHP and increased chances of PGD.^7^ Therefore, CBF settings should be applied to prevent coronary overflow but supply sufficient oxygen to the donor heart. The presented tool calculates this equilibrium CBF by balancing MVO_2_ and MDO_2_.

The tool computes CBF depending on the donor sex and BW, and on the SaO_2_, PaO_2_, and [Hb] in the perfusate. A minimal CBF of 500 ml·min^−1^ was selected, because preclinical studies have shown adequate perfusion, with less edema and better functional preservation, using this CBF.^6^ Yet, future studies should investigate whether CBF can be lowered even further.

### Improved Metabolic Assessment

Next to CBF optimization, the tool aids in the assessment of donor heart performance on ESHP. Currently, this assessment is based on lactate trends in the perfusate, with increasing lactate being an indicator to reject the heart for transplantation.^2^ However, several studies questioned the predictive value of lactate for this purpose.^8^ Alternatively, MVO_2_ and ΔPO_2_ trends better correlate with cardiac function^5,8^ and could thus provide additional markers of metabolic function on ESHP. However, computation of MVO_2_ is currently not performed on the OCS. The presented tool automatically evaluates the balance between MVO_2_ and MDO_2_ and recalculates the optimal CBF, while incorporating CVR^8^ and ΔPO_2_ as additional markers of oxygen handling.^5^

### Limitations

The CBF calculation in this study is based on the estimation of HW from donor sex and BW, whereas actual HW and CBF are dependent on real-world variations in cardiac anatomy. Measurement of the actual HW before cardiac instrumentation on the OCS could improve this. Also, the CBF calculation is based on assumptions derived from *in vivo* physiology. However, PO_2_ is generally higher on the OCS compared with *in vivo*, which results in excessive MDO_2_ on the OCS.^5^

### Future Perspectives

To date, the tool has been developed and tested in our own center. As a next step, we plan to conduct a clinical study to evaluate whether transplantation procedures using the tool result in reduced myocardial edema formation and improved clinical outcomes, compared with conventional OCS runs. In this study, HW will be measured before and after connection to the OCS machine, and the rate of mechanical circulatory support post-transplantation will be assessed.

## Conclusions

We present a novel tool that personalizes CBF and gives real-time feedback on oxygen handling for hearts on subnormothermic ESHP. Coronary blood flow is calculated from donor and procedural characteristics, potentially leading to reductions in myocardial edema formation during future ESHP procedures. In addition, the tool provides additional metabolic assessment parameters, next to lactate, providing a more comprehensive interpretation of graft performance. The question remains if application of this tool in clinical practice could improve transplantation outcomes by reducing the chances of PGD.

## Supplementary Material
