# Supplementary material for: A Smart Computational Tool for Personalized Coronary Blood Flow Settings During Normothermic Ex Situ Heart Perfusion
Source: ASAIO J. 2025 Feb 20;71(6):e90–3. doi: 10.1097/MAT.0000000000002394 (PMC12122086; doi:10.1097/MAT.0000000000002394)
Supplement: Supplementary file 1 [file mat-71-e90-s001.pdf]

A Smart Computational Tool for Personalized Coronary Blood Flow Settings during Normothermic Ex-Situ Heart Perfusion

Supplementary Materials

A. Supplementary Equations

B. Supplementary Table 1

C. Mathematical derivation of final formula

A. Supplementary Equations

Supplementary Equations 1 and **2.** Formulas to calculate heart weight from male (HW_M) and female donors (HW_F) based on body weight (BW).

$$HW_F = 2.153 \times BW + 104.6$$ (**Suppl. Eq. 1**)[1]

$$HW_M = 2.492 \times BW + 141.2$$ (**Suppl. Eq. 2**)[2]

Supplementary Equation 3. Normalization formula.

$$CBF_{norm} = (max_{value} - min_{value}) \times \frac{CBF - \min(CBF)}{\max(CBF) - \min(CBF)} + min_{value}$$ (**Suppl. Eq. 3**)

[1] Molina DK, DiMaio VJ: Normal Organ Weights in Women: Part I-The Heart. *Am J Forensic Med Pathol* 36 (3): 176-81, 2015.
[2] Molina DK, DiMaio VJ: Normal organ weights in men: part I-the heart. *Am J Forensic Med Pathol* 33 (4): 362-7, 2012.

B. Supplementary Table 1.

Extreme values used for normalization of personalized CBF formula. Values are based on transplant experience in the Erasmus MC[3].

BW = body weight. Hb = hemoglobin concentration. PaO_2 = partial arterial pressure of oxygen. SaO_2 = arterial oxygen saturation.

	**Minimum**	**Maximum**
Hb	5 g/dL	15 g/dL
PaO_2	60 kPa	87 kPa
SaO_2	98%	100%
BW_{male}	69 kg	100 kg
BW_{female}	60 kg	90 kg

[3] Jorik H. Amesz, Sanne J.J. Langmuur, Mark F.A. Bierhuizen, Dwight Dumay, Pieter C. van de Woestijne, Jelena Sjatskig, Lisa E. Sluijter, Dirk J. Duncker, Olivier C. Manintveld and Yannick J.H.J. Taverne, Myocardial Oxygen Handling and Metabolic Function of Ex-Situ Perfused Human Hearts from Circulatory Death Donors, JHLT Open, (2024) doi: https://doi.org/10.1016/j.jhlto.2024.100159

C. Mathematical derivation of final formula

**Step 1:**<br><br>Substitute max_{value} and min_{value} for coronary blood flow with 800 $mL \cdot min^{-1}$ and 500 $mL \cdot min^{-1}$.<br><br>$$= (800 - 500) \times \frac{CBF - \min(CBF)}{\max(CBF) - \min(CBF)} + 500$$
**Step 2:**<br><br>Substitute general CBF formula from Equation 3.<br><br>$$= 300 \times \frac{\frac{HW}{1.34 \times [Hb] \times SaO_2 + 0.0031 \times PaO_2} \times \frac{MvO_2\ per\ HW}{OER} - \min(CBF)}{\max(CBF) - \min(CBF)} + 500$$
**Step 3:**<br><br>Substitute MVO_2 per HW with 10% and OER with 80%.<br><br>$$= 300 \times \frac{\frac{HW}{1.34 \times [Hb] \times SaO_2 + 0.0031 \times PaO_2} \times \frac{0.10}{0.80} - \min(CBF)}{\max(CBF) - \min(CBF)} + 500$$
**Step 4:**<br><br>Subsitute HW with Supplementary Equations 1 and 2 (female given as example here).<br><br>$$= 300 \times \frac{\frac{2.153 \times BW + 104.6}{1.34 \times [Hb] \times SaO_2 + 0.0031 \times PaO_2} \times 12.5 - \min(CBF)}{\max(CBF) - \min(CBF)} + 500$$
**Step 5:**<br><br>Calculate min(CBF) based on data from Supplementary Table 1 by dividing the lowest HW by the highest [Hb], PaO_2 and SaO_2, and max(CBF) by dividing the highest HW by the lowest [Hb], PaO_2 and SaO_2 (female given as example here).<br><br>$$= 300 \times \frac{\frac{2.153 \times BW + 104.6}{1.34 \times [Hb] \times SaO_2 + 0.0031 \times PaO_2} \times 12.5 - \frac{234}{1.34 \times 15 \times 1 + 0.0031 \times 650} \times 12.5}{\frac{298}{1.34 \times 5 \times 0.98 + 0.0031 \times 450} \times 12.5 - \frac{234}{1.34 \times 15 \times 1 + 0.0031 \times 650} \times 12.5} + 500$$<br><br>$$= 300 \times \frac{\frac{2.153 \times BW + 104.6}{1.34 \times [Hb] \times SaO_2 + 0.0031 \times PaO_2} \times 12.5 - 133}{333} + 500$$
